# A decade of clinical experience with extra-adrenal paragangliomas of retroperitoneum: Report of 67 cases and a literature review

**DOI:** 10.4103/0974-7796.62919

**Published:** 2010

**Authors:** Jin Wen, Han-Zhong Li, Zhi Gang Ji, Quan Zhong Mao, Bing Bing Shi, Wei Gang Yan

**Affiliations:** Department of Urology, PeKing Union Medical College Hospital, Chinese Academy of Medical Sciences and PeKing Union Medical College, Beijing 100730, China

**Keywords:** Extra-adrenal paraganglioma, retroperitoneal tumor, diagnosis, treatment

## Abstract

**Object:**

The purpose was to highlight the diagnosis and treatment of extra-adrenal para-gangliomas, which often causes catecholamine hypersecretion and hypertension.

**Methods:**

67 cases of extra-adrenal paraganglioma of retroperitoneum proven pathologically from 1999 to 2009 were reviewed and studied after operation. Endocrine secretion examinations, B-US, CT, MRI, 131-MIBG, octreotide and hands microcirculation inspection were used to diagnose the disease.

**Results:**

All patients underwent successful surgical resection of the tumors, which proved to be paragangliomas. They were from 3 cm to 25 cm in size. Almost all of them were diffusely positive for cgA, syn, NSE and s-100 by immunohitochemical staining. There were nine cases assayed malignant paraganglioma by the follow-up.

**Conclusions:**

131-MIBG and octreotide have high sensitivity and accuracy in diagosing extra-adrenal paraganglioma. Surgical treatment should be carried out on the basis of correct drug preparation of α-receptor blocker, such as prazosin and phenoxybenzamine. Complete surgical excision is the treatment of choice for extra-adrenal paragangliomas as well as recurrent or metastatic disease, which could be resected laparoscopically. Intimate lifelong follow-up is necessary and important.

## INTRODUCTION

Extra-adrenal retroperitoneal paragangliomas (PGLs) arise from dispersed paraganglia that tend to be symmetrically distributed in close relation to the aorta and sympathetic nervous system. They are rarely encountered in every day surgical practice. Meanwhile, Extra-adrenal retroperitoneal paragangliomas are rare tumors causing considerable difficulty in both, diagnosis and treatment. They can be unicentric or multicentric, tend to be locally invasive and, therefore have a high incidence of local recurrence. Histological and immunological phenotype of silent extra-adrenal retroperitoneal paragangliomas are no significant difference with functional ones. In this article, we reviewed the experience in our hospital of this uncommon tumors to highlight the diagnosis and treatment of extra-adrenal paragangliomas.

## METHODS

This was a retrospective single-institutional study. We identified 67 adult patients (33 women and 34 men; mean age, 41.6 years; age range, 15–61 years) with extra-adrenal PGLs of retroperitoneum between 1999 and 2009 in PeKing Union Medical College Hospital (PUMCH). The number of silent extra-adrenal paraganglioma of retroperitoneum was 18 % of all 67 retroperitoneal paraganglioma resected by operation during this period of time in PUMCH. Patients with silent extra-adrenal retroperitoneal PGLs were older than those of fuctional tumors with more tumor diameters [Table 1]. The clinical behavior of extra-adrenal PGLs of retroperitoneum is not determined by tumor location, that is, symptoms and signs do not depend on the site of origin. Malignancy is defined by the existence of metastasis rather than by histology.

**Table 1:** Presentations of all patients with extra-adrenal retroperitoneal paragangliomas (PGLs) in the study

Item	Silent extra-adrenal retroperitoneal PGLs	Functional extra-adrenal retroperitoneal PGLs
Number of cases	12	55
Gender (male/female)**	4/8	30/25
Mean age (years old)*	42.8	40.3
Tumor diameters*	3-20 cm	4-17 cm
Location (near renal hilum/in para-aorta)**	5/7	24/31

* *P* < 0.05,

** *P* > 0.05

Endocrine secretion examinations, B-US, CT, MRI, 131I-MIBG, and octreotide were performed for preoperative diagnosis. US can be a simple and effective diagnostic method. The diameter of adrenal masses as measured by US correlates highly with mass diameter measured by CT. FDG-PET has been suggested for the characterization of adrenal masses in patients. At the same time urine UFC, serum F, blood ACTH, size of the dose dexamethasone suppression test, blood renin - blood vessels angiotensin - aldosterone, and other tests were used to rule out other diseases. Hands microcirculation inspection was used to understand systemic microcirculation. Preoperative management is aimed at prevention of possible catecholamine-induced complications like hypertensive crisis, cardiac arrhythmias, pulmonary oedema and cardiac ischemia due to manipulation of the tumor, and especially to prevent hypotensive crises after tumor removal. We prefer to use an adrenoceptor blocking agent for 2–4 weeks.

The medical case records were also reviewed. Pathological evaluation was performed with HE staining and S-P immunohistochemical test by two pathologists. Positive CgA, Syn, NSE staining was diffuse cytoplasmic brown granules, while positive S-100 staining was in the nucleus and the cytoplasm.[1]

## RESULTS

Hypertension were observed in 55 functional extra-adrenal retroperitoneal paragangliomas before operation, who were admitted to hospital because of headache, palpitations and diaphoresis.

While silent tumors were admitted to hospital by routine physical examination or tumor-related pain, no abdominal mass was palpable in all patients. Six patients had been found with familial history.

The tumors were from 3 cm to 25 cm in size. MSCT shows a round or similarly round retroperitoneal tumor with inner central hemorrhage and necrosis. Tumors show obvious intensification after injection of contrast medium. [Figures 1A–B]. All patients underwent successful surgical resection of the tumors, which proved to be paragangliomas. Almost all of them were diffusely positive for cgA, syn, NSE and s-100 by immunohitochemical staining.

**Figure 1a:**
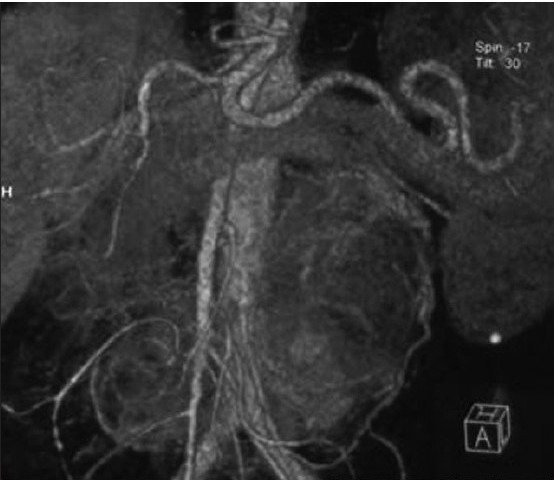
MSCT shows a round or similarly round retroperitoneal tumor with inner central hemorrhage and necrosis. Tumors show obvious intensification after injection of constrast medium. a) Multiple extra-adrenal retroperitoneal paragangliomas. Coronal contrastenhanced reformatted CT scan shows two lobulated soft-tissueattenuation masses (arrows) above the aortic bifurcation

**Figure 1b:**
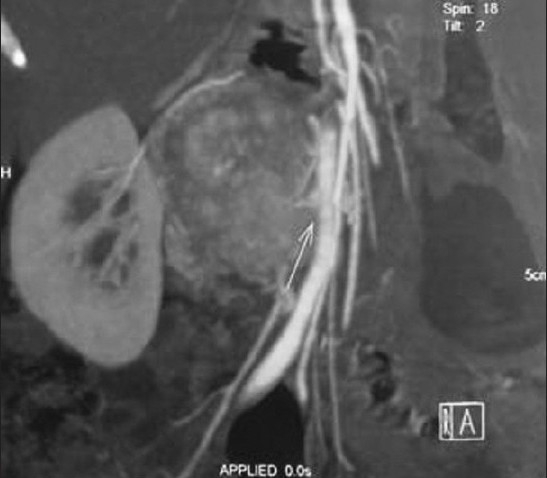
MSCT shows a round or similarly round retroperitoneal tumor with inner central hemorrhage and necrosis. Tumors show obvious intensification after injection of constrast medium. b) Coronal contrast-enhanced reformatted CT scan shows one lobulated soft-tissue-attenuation masses (arrows) near the right renal hilum

The cut surface of the tumor showed integrated amicula and abundant blood supply. There was a large area remote hemorrhage and necrosis inside of tumor [Figures 2A–B]. Under light microscope, the tumor possessed an evident fibrous capsule. The tumor cells were same in size with distinct boundary [Figure 3]. Neoplastic cells were diffusely positive for cgA, syn, NSE and s-100 by immunohitochemical staining. These chief cells generally had central nuclei lacking atypia. Up to now, no widely accepted pathological criteria exist for differentiating between benign and malignant PGLs. Metastatic disease remains the only irrefutable proof of malignancy. There were 9 cases assayed malignant paraganglioma by follow-up.

**Figure 2a:**
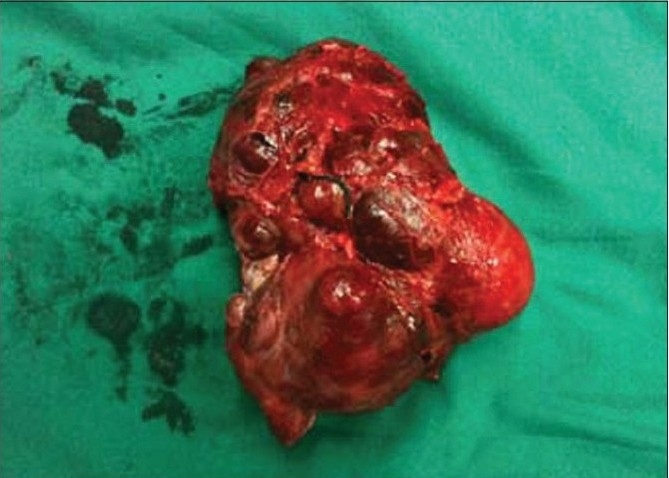
Tissue sample acquisition; a) The speciman of paraganglioma after operation showed integrated amicula and abundant blood supply

**Figure 2b:**
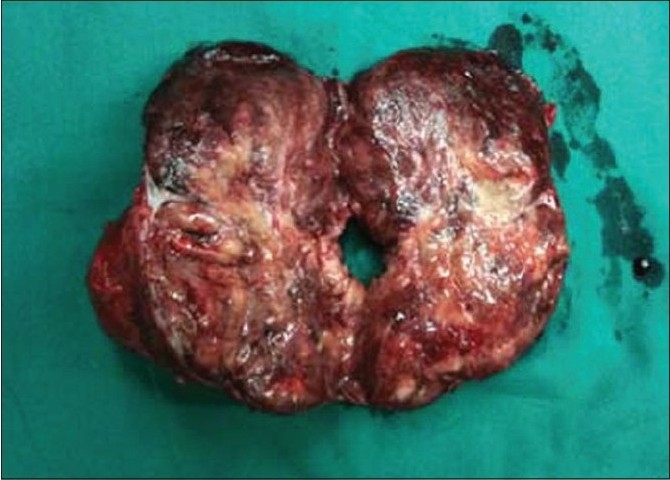
Tissue sample acquisition; b)There was a large area remote hemorrhage and necrosis inside of tumor

**Figure 3:**
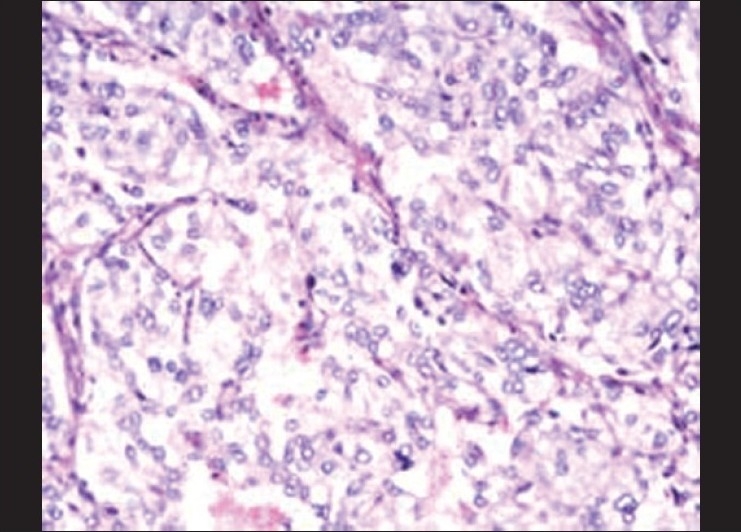
Under light microscope, the tumor cells were same in size with distinct boundary. Tumor was an alveolar-like structure with many vascular septa. Mitotic structures and nuclear atypia are infrequent

## DISSCUSSION

Extra-adrenal PGLs of retroperitoneum are neoplasias of the chromaffin cells which are located in the para-aortic sympathetic chain, aortic bifurcation.[2] They often manifest themselves by symptoms of episodic freeing of catecholamines, such as high blood pressure, migraines, sweating and palpitations. They are extremely rare because incidence of all extra-adrenal PGLs is only 2–8 per million, which occur either sporadically or as part of a hereditary syndrome. The classical familial syndromes is associated are multiple endocrine neoplasia type 2 (RET mutations), von Hippel Lindau disease (VHL mutations), hereditary PGL/pheochromocytoma syndromes (SDHx mutations) and rarely neurofibromatosis type 1 (NF1 mutations).[3] Although there have been several reports of patients with the tumors of considerable size, yet normal plasma and urine catecholamine concentrations,[4] few reports have described the natural history of the tumor. It is reported silent extra-adrenal PGL of retroperitoneum is probably due to mutations of the gene for succinate dehydrogenase-B (SDHB).[5]

Almost all the patients in our study were found PGLs of retroperitoneum by US in physical examination according to medical records. However, the US depends to a large extent on operator skills. Furthermore, obesity and overlying gas are obstacles for the visualization. Unsurprisingly, the US does not detect tumors with the same sensitivity as CT or MRI. The tumors usually appear as rounded or oval masses with a similar density to the liver on unenhanced CT. Larger lesions may show a cystic component due to central necrosis or hemorrhage. Calcification is present in some cases. Owing to their hyper-vascularization, the tumors usually exhibit intense enhancement. The tumors are generally characterized by low T1 and bright T2 signal intensities. Central necrosis is frequently observed. There are no signal changes from out-of-phase to in-phase images. For the localization and identification of PGLs of retroperitoneum, the radio-pharmaceuticals 131I-MIBG and 111in-octreotide have been most commonly used. The sensitivity of 131I-MIBG for detecting PGLs ranges between 80 and 90%, with a specificity of 90–100%.

Most malignant tumors show an enhanced glycolytic metabolism with increased up-take of deoxyglucose that can be visualized by PET using18F-2-fluoro-d-deoxyglucose (FDG). However to date, there are insufficient data to justify the use of PET to diagnose clinically silent extra-adrenal PGLs of retroperitoneum outside clinical studies. We are engaged in the relevant research with help of PET center of our hospital.

Hypertension and urinary catecholamine was usually elevated in extra-adrenal PGL of retroperitoneum. The measurement of plasma catecholamines is not recommended because this method has poor sensitivity and specificity, which often leading to false-positive results. Urine metanephrines have a higher specificity, receiver operating characteristic curves revealed a better test performance than other biochemical tests.[6] Special attention should be given to acetaminophen use, which interferes with assays and is a source of false-positive testing .Although chromogranin A is not specific for pheochromocytoma , its evaluation can be useful. The level of chromogranin A correlates with tumor mass.[7] Thorough preoperative pharmacological preparation, attentive intraoperative monitoring and aggressive surgical therapy all have an important role in achieving the safest and most successful outcome .Operative mortality is down to below one percent with adequate pharmacological Preparation.[8]

Almost all our patients were performed a complete surgical resection without hypertensive crisis, who were took α-receptor blocker for 2-4 weeks preoperatively. Among 12 silent extra-adrenal PGLs of retroperitoneum, four cases were operated without pharmacological preparation, two of whose blood pressure were observed rising close up to 27/16 kpa transiently. This shows that silent extra-adrenal retroperitoneal paragangliomas is nonfunctional relatively, In the practical clinical work, it is possible to result in severe consequences if the preoperative awareness is not enough. Randomized controlled trials for preoperative drug treatment are lacking temporarily.

Surgery remains the mainstay of treatment of extra-adrenal PGLs of retroperitoneum. However, it must be born in mind that the "silent" extra-adrenal PGLs of retroperitoneum is silent ones relatively in the handling of these tumors. Carrying out of physical maneuvers on the tumor and the employment of drugs that free catecholamines can induce hypertensive crises. Once the diagnosis and drug preparation are accomplished, attempt should be made to perform a complete surgical resection. Resection is often challenging as these highly vascular tumors are located near multiple vital blood vessels.

Initially, Almost all PGLs of retroperitoneum (10/11) were performed via the open trans-abdominal route in our hospital, especially before 2003. With the development of laparoscopic technique, we applied the technique to resect PGLs of retroperitoneum (32/56) successfully. Initial indications were limited to small tumors due to concerns about bleeding, the safety of removing tumors (especially under carbon dioxide insufflation, which might theoretically trigger a hypertensive crisis). As we gained experience, indications for laparoscopic resection expanded to include large tumors more than 8 cm [Figures 2A–B]. We also developed the technique with reduction of mean operative time and mean blood loss with no mortality and major complications. The laparoscopic approach may have advantages over the open approach when performed by a surgical team experienced in advanced laparoscopic techniques, including decreased postoperative pain, reduced time to return of bowel function, decreased length of hospital stay, and the potential for earlier return to work. When residual tumor cannot be resected, medical therapy for symptomatic relief is preferred, radiotherapy and chemotherapy have limited effectiveness.

Extra-adrenal retroperitoneal PGLs have a more aggressive course than their adrenal counterparts, which are called pheochromocytomas. The prognosis of PGLs can be very difficult to predict. However, their anatomical location may influence their biological behavior: carotid body PGLs have a relatively low rate of malignant behavior while retroperitoneal PGLs tend to have a higher incidence of malignant transformation. Approximately 20–42% of PGLs metastasize, compared to only 2–10% of adrenal pheochromocytomas. Dissemination occurs both lymphatically and hematogenously, with the most common sites of metastasis being the regional lymph nodes, bone, liver, and lung. Distant metastasis to bones, lymph nodes and lungs has been reported as a unique feature of the metastatic spread of extra-adrenal retroperitoneal PGLs.[9] Diagnosis of malignant PGLs is based on evidence of extensive local invasion , or more reliably on documentation of metastasis to one or more sites where nonchromaffin tissue is not normally present.[10] According to current WHO criteria, local invasion alone does not define malignancy because it is not always present in tumors that metastasize.[11]

Up to 36% of the extra-adrenal sympathetic PGLs were malignant. Consequently, not only metastasis but also local invasion should be carefully evaluated when making a diagnosis of sympathetic PGL. There were nine cases which assayed malignant PGL by follow-up. A total of six cases with paraganglioma infiltrating the surrounding lymph nodes were diagnosed as malignant tumors, two were found relapsed 2–4 years after operation; one case of bone metastasis occurred five years after operation. These may indicate the disease has a tendency to relapse and may be multi-center results. Therefore, intimate follow-up is necessary and very important. Treatment with therapeutic doses of 131I-MIBG or combination chemotherapy (cyclophosphamide, vincristine, and darcabazine) may induce (partial) responses to malignant PGLs.[12]

Taken together, A heterogeneous, hypervascular, retroperitoneal mass with areas of necrosis with typical clinical setting of headaches, hypertension and tremor, is highly predictive of the presence of an extra-adrenal PGLs. 131I-MIBG and octreotide have high sensitivity and accuracy in diagnosing silent extra-adrenal PGL. Once the correct diagnosis is acquired, surgical treatment should be carry out after using alpha-adrenergic blockade for 2–4 weeks in order to prevent and treat a syndrome of possible intra-operative catecholamine release. Tumors should be resected laparoscopically, if possible. Future efforts should be directed toward obtaining a larger database to define the true natural history of extra-adrenal retroperitoneal PGLs. More prospective clinical trials are needed to provide the most reliable evidence regarding the management of patients.
